# Imaging of Hip Pain: From Radiography to Cross-Sectional Imaging Techniques

**DOI:** 10.1155/2016/6369237

**Published:** 2016-01-13

**Authors:** Fernando Ruiz Santiago, Alicia Santiago Chinchilla, Afshin Ansari, Luis Guzmán Álvarez, Maria del Mar Castellano García, Alberto Martínez Martínez, Juan Tercedor Sánchez

**Affiliations:** ^1^Radiology Department, Hospital of Traumatology, Carretera de Jaen, S/N, 18014 Granada, Spain; ^2^Radiology Department, Ciudad Sanitaria Virgen de las Nieves, Avenida de las Fuerzas Armadas 2, 18014 Granada, Spain; ^3^Radiology Department, North Tyneside General Hospital, Rake Lane, North Shields NE29 8NH, UK; ^4^Orthopedic Department, Hospital of Traumatology, Carretera de Jaen, S/N, 18014 Granada, Spain

## Abstract

Hip pain can have multiple causes, including intra-articular, juxta-articular, and referred pain, mainly from spine or sacroiliac joints. In this review, we discuss the causes of intra-articular hip pain from childhood to adulthood and the role of the appropriate imaging techniques according to clinical suspicion and age of the patient. Stress is put on the findings of radiographs, currently considered the first imaging technique, not only in older people with degenerative disease but also in young people without osteoarthritis. In this case plain radiography allows categorization of the hip as normal or dysplastic or with impingement signs, pincer, cam, or a combination of both.

## 1. Introduction

In the last years, advancements in knowledge of biomechanics and hip joint functional anatomy, as well as improvements in arthroscopy procedures and refinements of imaging techniques, have widened the spectrum of diagnoses causing pain around the hip joint.

Radiologists, as part of the diagnostic team, have to know the appropriate use of different imaging techniques in order to reach an accurate diagnosis without delaying patient management.

## 2. Causes of Hip Pain

Causes of pain around the hip joint may be intra-articular, extra-articular, or referred pain from neighboring structures, such as sacroiliac joint, spine, symphysis pubis, or the inguinal canal [1].

Intra-articular causes include the following: labral tears, chondromalacia, degenerative changes, intra-articular bone injury, ligamentum teres rupture, arthritis (inflammatory, infectious, etc.), and synovial proliferative disorders.

Extra-articular causes include the following: tendinopathy, bursitis, iliotibial band syndrome, muscle injury, and piriformis syndrome.

This editorial review is going to focus mainly on intra-articular causes of hip pain.

## 3. Hip Pain Imaging: Need for Clinical Correlation

Imaging of the hip needs to be complementary to the clinical history and physical examination because it is well known that imaging findings do not always correlate with the presence of pain and vice versa.

Clinical tests are adapted to identify the source of pain as intra-articular or extra-articular. The flexion-abduction-external rotation (FABER), internal range of motion with overpressure (IROP), and scour tests show sensitivity values in identifying individuals with intra-articular pathology ranging from 0.62 to 0.91 [2].

In the next subheadings, we are going to describe the main indications and role of different imaging modalities (X-ray, magnetic resonance imaging (MRI), computed tomography (CT), ultrasound, and scintigraphy) in studying intra-articular causes of hip pain.

## 4. X-Ray: A Basic Approach

Radiographs are currently useful not only in older patients in whom osteoarthritis of the hip is suspected but also in younger patients without osteoarthritis, who are being evaluated for femoroacetabular impingement (FAI) or hip dysplasia.

**Figure 1:**
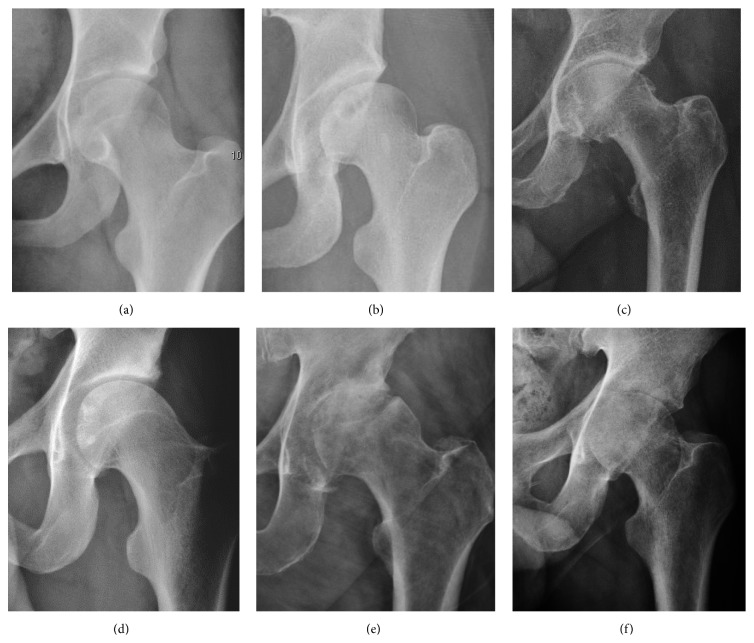
Radiography in normal hip (a), hip dysplasia (b), pincer impingement type (c), and cam (d). Hip in osteoarthritis (e) and septic arthritis (f).

Plain radiography allows us to categorize the hip as normal or dysplastic or with impingement signs (pincer, cam, or a combination of both). Besides these, pathologic processes like osteoarthritis, inflammatory diseases, infection, or tumors can also be identified (Figure 1).

### 4.1. X-Ray in Pediatric Age

Radiographs of infants should be obtained with the pelvis in neutral position with the lower limbs held in neutral rotation and slight flexion. Reliability of measurements increases if indicators of pelvic alignment are taken into account. Tönnis introduced a quotient of pelvic rotation by dividing the horizontal diameter of the obturator foramen of the right side and that of the left. In neutral rotation the ratio is 1 but is considered to be acceptable when it is between 0.56 and 1.8. The symphysis os-ischium angle of Tönnis evaluates the pelvic position in the sagittal plane. Lines are drawn from the highest point of the ischium to the most prominent point of the symphysis, joining at the inside of the pelvis. The range of normal values is from 90 to 135° and is related to the infant's age [3].

Despite the widespread of ultrasound, pelvis radiographs are still frequently used to diagnose and/or monitor DDH or for assessing other congenital conditions or bone tumors [4]. The Tönnis method is the most widely used radiographic system to classify DDH [5]. It relies on the presence of the femoral head ossification center. Because eccentric position or delayed appearance of the ossific nucleus is a common finding in DDH, a new radiographic classification system has been developed by the International Hip Dysplasia Institute (IHDI), which uses the mid-point of the proximal femoral metaphysis as a reference landmark [6].

**Figure 2:**
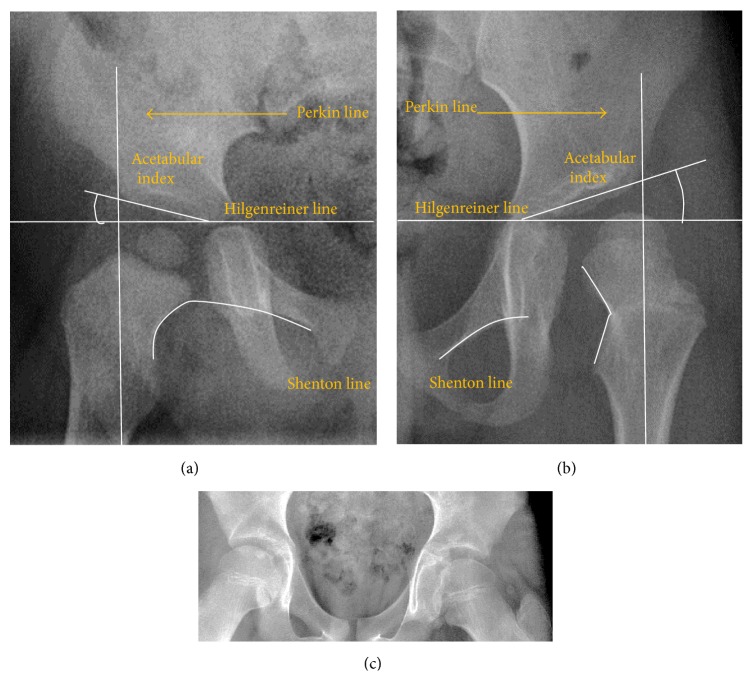
Radiological measurements in pediatric normal (a) and dysplastic hip (b). (c) AP view of a patient with left hip effusion secondary to trauma showing widening of the medial joint space.

The most useful lines and angles that can be drawn in the pediatric pelvis assessing DDH are shown in Figure 2.

(A) *Hilgenreiner Line*. It is considered a basal line joining the top aspect of the triradiate cartilages. This line is used to measure the acetabular angle and as a reference for Perkin line.
(B) Perkin line is perpendicular to Hilgenreiner line, touching the lateral margin of the acetabulum. This leads to four quadrants and a normal femoral head has to be located in the inferomedial quadrant. We can measure the lateral displacement of the femoral head with regard to the Perkin line by dividing the width of the head that crosses the Perkin line by the diameter of the head. The value for patients under 3 years must be 0 and in older children this ranges from 0 to 22%.
(C) Shenton line is a continuous arc drawn from the inner edge of the femoral neck to the superior margin of the obturator foramen. This should be smooth and undisrupted; otherwise it may indicate a fracture or hip dysplasia.
(D) The acetabular index measures the acetabular roof slope. It is the most useful measure of acetabular dysplasia until 6 years of age. It is formed between Hilgenreiner line and the acetabular roof. In newborns, values of 26° ± 5° in males and 30° ± 4° in females are considered normal. Gradually this angle becomes smaller, with a mean value of 18° ± 4° in males and 20° ± 3° in females at 1 year of age [7].
(E) The medial articular joint space is measured between the medial border of the femoral head or neck (when epiphysis is not ossified) and the acetabular platform. Normal values range between 5 and 12 mm. Differences greater than 1.5 mm between the two sides are considered abnormal [8].

**Figure 3:**
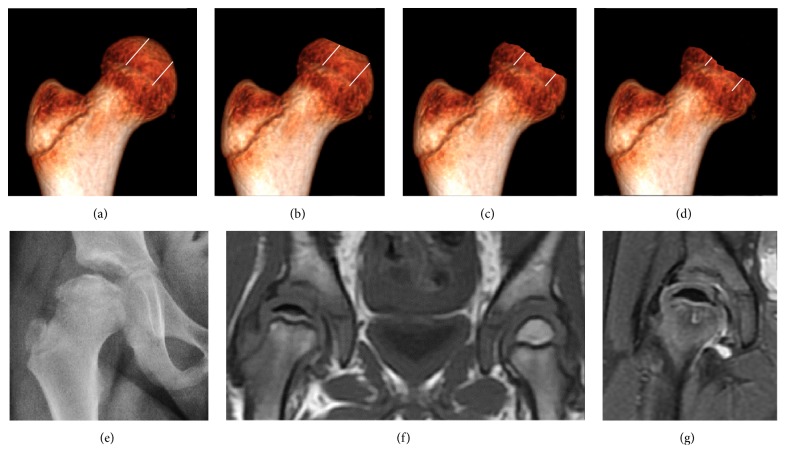
Herring lateral pillar classification. Groups A (a), B (b), B/C (c), and C (d). AP radiograph (e), coronal T1 (f), and PD fat sat (g) weighted images showing loss of fat signal of the epiphysis, edema, and cyst formation in femoral metaphysis in a grade C Perthes disease.

Most cases of Legg-Calvé-Perthes disease (LCPD) develop between the ages of 4 and 10 years (Figure 3). Classification of its severity can be assessed by radiographs. Herring or lateral pillar classifications and the patient's age strongly correlate with the outcome [9]. In Group A, which has a better prognosis, there are no loss of height in the lateral third of the femoral head and little density changes; in Group B, there is lucency and lateral height loss of less than 50%; and in Group C, the most severe form, there is more than 50% loss of lateral height. Group B/C is considered when the loss of lateral pillar height is at 50% [10]. Patients who are over the age of 8 years at the time of onset and have a hip in the lateral pillar B group or B/C border group have a better outcome with surgical treatment than they do with conservative treatment. Group B hips in children who are less than 8 years at the time of onset have a very favorable outcome unrelated to the treatment, whereas Group C hips in children of all ages usually have poor outcome unrelated to the treatment [11].

**Figure 4:**
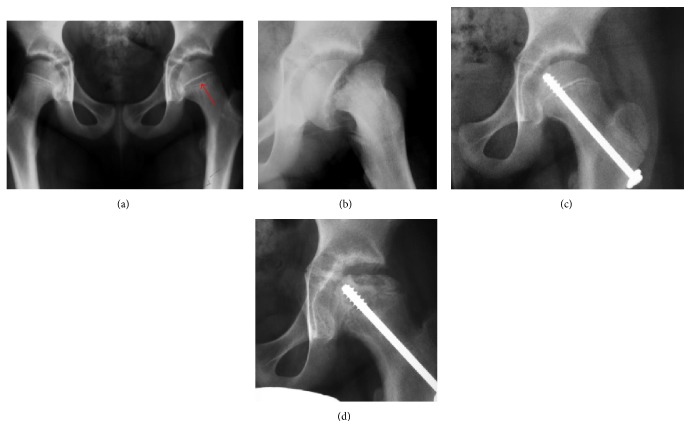
(a) X-ray of a 10-year-old child with left hip pain. It was considered normal at emergency despite the widening of the left physis (arrow). Two weeks later epiphysiolysis was evident (b). Despite appropriate surgical reduction (c) osteonecrosis developed and femoral head collapsed 1 month later (d).

Slipped Capital Femoral Epiphyses (SCFE) usually affect 11- to 14-year-old patients (Figure 4). Radiographs may show widening and irregularity of the physis and posterior inferior displacement of the capital femoral epiphysis. On the AP view Klein's line, tangent to the lateral aspect of the femoral neck, does not intersect the femoral head indicating that it is displaced. SCFE may compromise the blood supply to the femoral head and cause avascular necrosis, mainly when there is instability between the fragments [12].

### 4.2. X-Ray in Adult Age

**Figure 5:**
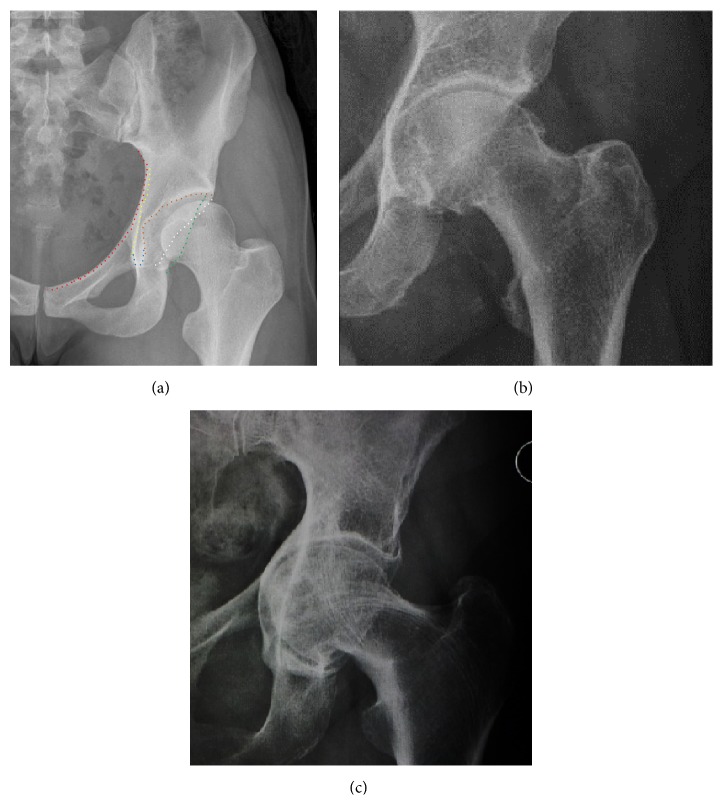
(a) Iliopectineal line (red), ilioischial line (yellow), tear drop (blue), acetabular fossa (brown), and anterior (white) and posterior (green) wall of the acetabuli showing mild upper crossover sign. (b) Coxa profunda. (c) Protrusio acetabuli.

In the adult hip there are important landmarks to be recognized on plain film radiographs (Figure 5):

- Iliopectineal or iliopubic line is formed by the arcuate line of the ilium and the superior border of the superior pubic ramus up to the pubic symphysis. It conforms to the inner margin of the pelvic ring and it is part of the anterior column of the acetabulum.
- The ilioischial line of Köhler begins at the medial border of the iliac wing and extends along the medial border of the ischium to end at the ischial tuberosity. It is part of the posterior column of the acetabulum.
- *Acetabular Floor*. In normal conditions the floor of the acetabular fossa is lateral to the ilioischial line by 2 mm in men and 1 mm in women. When the acetabular floor overlaps or overpasses the ilioischial line, the diagnosis of coxa profunda can be made. Nevertheless, coxa profunda had been found in 76% of asymptomatic hips, mainly in women. Therefore, this as an isolate criterion is not enough to make the diagnosis of pincer-type impingement [13]. A more severe condition is protrusio acetabuli, diagnosed when the femoral head overlaps or overpasses the ilioischial line (Figure 5).
- The teardrop represents a summation of shadows. Its medial aspect corresponds to the inner cortex of the pelvis and the lateral edge with the acetabular notch and the anteroinferior portion of the quadrilateral plate [14]. It is not present at birth but gradually develops due to pressure of the femoral head.

**Figure 6:**
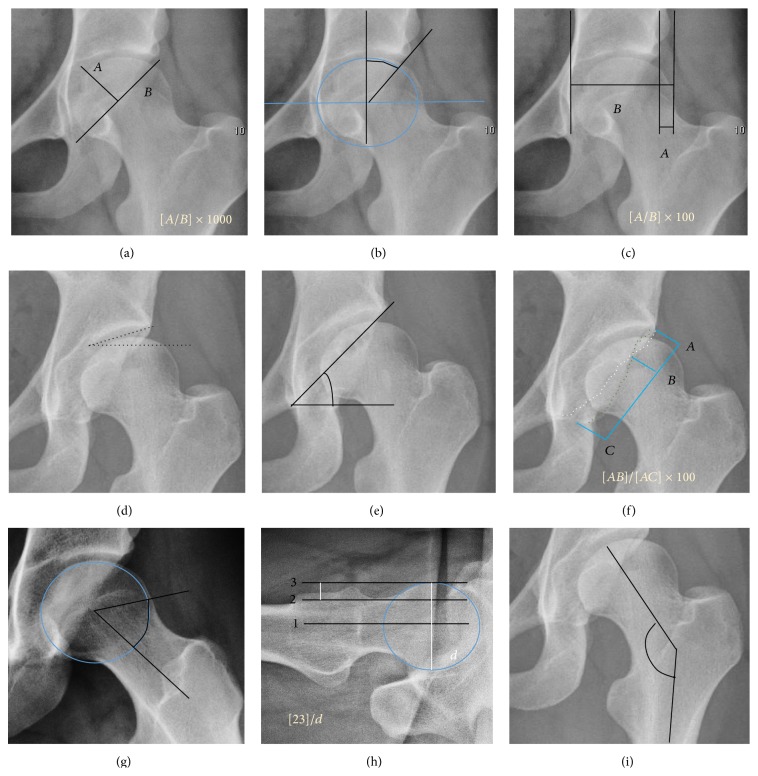
(a) Acetabular depth ratio. (b) Center-edge angle of Wiberg. (c) Femoral extrusion index. (d) Tönnis angle. (e) Sharp angle. (f) Crossing ratio. (g) Alpha angle measured in 45° Dunn view. (h) Offset percentage measured in cross-lateral view. (i) Cervical diaphyseal angle.

In the adult hip, normal joint space ranges from 3 to 5 mm and must be uniform. Values under 2 mm are consistent with joint space narrowing [15]. The most important measurements are detailed in Figure 6 and Table 1.

Acetabular depth value under 250 characterizes the dysplastic hip [16].

In normal conditions the acetabulum covers 75% of the femoral head. This coverage can be determined by three different measurements: lateral center-edge angle of Wiberg, anterior center-edge angle, and femoral extrusion index. Femoral extrusion index measures the percentage of the femoral head that lies outside of the acetabular roof. This percentage must be inferior to 25% in adults.

Center-edge Wiberg's angle measures the superior-lateral coverage of the femoral head. It is useful in children older than 5 years and in adulthood. For children between 5 and 10 years the minimum normal value is 15°, and in adults it is about 20°, although after 55 years this minimum increases to 24° [17]. Values over 40° indicate overcoverage.

Anterior center-edge Lequesne's angle can be measured in a false profile view of the hip or in a sagittal CT scan. In this case the tangent line touches the anterior rim of the acetabulum. Values under 20° indicate undercoverage of the femoral head [18].

The acetabular slope can also be measured by different methods. The Tönnis angle quantifies the slope of the sourcil (the sclerotic weight-bearing portion of the acetabulum). Values over 10° are considered a risk factor for instability, while values under 0° are considered a risk factor for pincer impingement.

The Sharp angle is a global way to measure the acetabular slope. Angles over 45° are indicative of acetabular dysplasia.

Normal acetabulum is oriented in anteversion. Its value ranges from 15 to 20° in the equatorial plane of the acetabulum and decreases gradually towards the acetabular roof, where normal values range from 0 to 5°. Retroversion of the upper part of the acetabulum has been related with pincer type impingement. In radiography the presence of a “crossover sign” is produced when the posterior wall of the acetabulum crosses the anterior wall before reaching the acetabular roof. It is a sign of acetabular retroversion and it has been linked with overcoverage and pincer impingement. Nevertheless, this sign has been described in 6% of the normal population [19]. Therefore, more important than its presence is the percentage of crossing. This ratio is considered significant if it is over 20% [20].

Other signs associated with acetabular retroversion are the sciatic spine and posterior wall signs. The first one is considered positive when the sciatic spine is projected medial to the iliopectineal line in an AP radiography of the spine, indicating that it is not just the acetabulum but the whole hemipelvis that is twisted into retroversion. The second sign is considered positive when the posterior wall edge is medial to the center of the femoral head, indicating deficiency of the posterior wall.

In normal conditions there is a symmetric concave contour at the junction of the anterior and posterior profile of the femoral head and neck. Loss of this concavity or bone bulging may lead to cam type impingement. The degree of this deformity can be measured by the alpha angle. Although it can be measured in the cross-lateral view, the 45° Dunn view is considered more sensitive and the frog leg view more specific in determining pathologic values. Debate about which values are considered normal is still in progress. Based on the Copenhagen Osteoarthritis Study, a recent work defined three ranges of values for the *α*-angle: pathological (≥83° in men and ≥57° in women), borderline (69° to 82° in men, 51° to 56° in women), and normal (≤68° in men and ≤50° in women) [21].

The offset between the neck and femoral head can also be calculated in the lateral projection of the hip. A value of less than 10 mm is considered pathologic. The percentage is calculated by dividing the distance between the femoral head and the neck lines by the femoral head diameter. If this percentage is under 0.18 there is high probability of cam type impingement [22].

The angle formed between the femoral neck and femoral diaphysis ranges from 120° to 140°. Coxa valga is diagnosed if the angle is higher and coxa vara if the angle is lower than this normal range.

Although femoral version or torsion can be measured by radiographs, CT overcomes the inconsistencies demonstrated in the measurements made by biplane radiography [23].

In adults, one of the main indications for radiographs is the detection of osteoarthritic changes (Figure 1(e)). Nevertheless, radiographs usually detect advanced osteoarthritis that can be graded according to the Tönnis classifications. The grading system ranges from 0 to 3, where 0 shows no sign of osteoarthritis. Intermediate grade 1 shows mild sclerosis of the head and acetabulum, slight joint space narrowing, and marginal osteophyte lipping. Grade 2 presents with small cysts in the femoral head or acetabulum, moderate joint space narrowing, and moderate loss of sphericity of the femoral head. Grade 3 is the severest form of osteoarthritis, which manifests as severe narrowing of the joint space, large subchondral cyst with productive bone changes that may lead to deformity of the bone components of the joint [24], while secondary osteoarthritis due to calcium pyrophosphate deposition can be diagnosed when calcification of hyaline cartilage and fibrocartilage is detected [25].

There are other pathological conditions that can affect the hip joint and radiographs help to make the appropriate diagnosis. Acute bacterial septic arthritis can be diagnosed by radiographs when a fast regional osteoporosis and destructive monoarticular process develops (Figure 1(f)). In case of tuberculous or brucella arthritis it is manifested as a slow progressive process, and diagnosis may be delayed [26].

**Figure 7:**
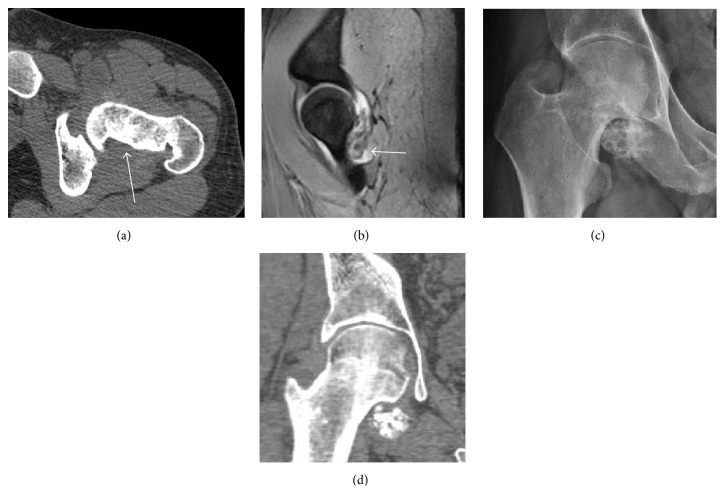
(a) Axial CT image of pigmented villonodular synovitis eroding the posterior cortex of the femoral neck. (b) Sagittal T2^*∗*^ gradient echo image showing a posterior soft tissue mass with hypointense areas secondary to hemosiderin deposition. (c) X-ray film and computed tomography (d) in synovial chondromatosis.

Synovial chondromatosis can be confidently diagnosed by X-ray when calcified cartilaginous chondromas are seen. However, other synovial proliferative processes, such as pigmented villonodular synovitis, require MRI for accurate diagnosis, although noncalcified synovitis can be suspected in radiographs by indirect signs, such as soft tissue swelling and/or erosions in the femoral head, femoral neck, or acetabulum (Figure 7) [27].

**Figure 8:**
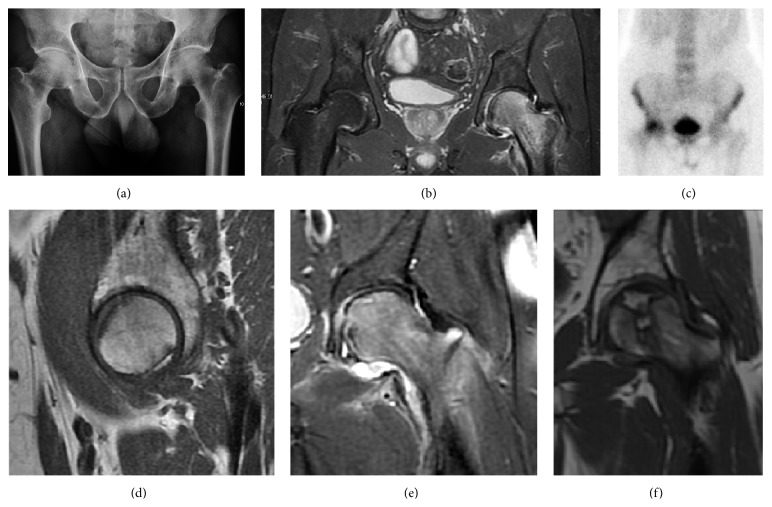
(a) X-ray of a patient with transient osteoporosis of the left hip showing osteoporosis. (b) Coronal stir imaging showing diffuse edema. Scintigraphy (c), sagittal T1 (d), and coronal PD fat sat (e) of a patient with a subchondral fracture of the femoral head with convex shape to the articular surface. Coronal T1 (f) of a patient with avascular necrosis of the femoral head.

Radiological signs of transient osteoporosis of the hip include localized osteoporosis of the femoral head and neck (Figure 8). Nevertheless, final diagnosis has to be made with MRI to differentiate it from avascular necrosis and from insufficiency or stress fractures of the femoral head or neck. In case of AVN, radiographs can only demonstrate delayed or advanced signs. Staging according to Ficat classification ranges between normal appearance (stage I), slight increased density in the femoral head (stage II), subchondral collapse of the femoral head with or without “crescent” sign (stage III), and advanced collapse with secondary osteoarthritis (stage IV) [28]. In the case of stress or insufficiency fractures X-ray sensitivity has been proven to be much lower than MRI, which is currently the gold standard [29].

## 5. Magnetic Resonance 

Many pathological conditions of the hip are detected early by MRI due to its high soft tissue resolution and sensitivity. Its accuracy in studying acute hip pain in children has proved to be superior to ultrasound and plan film radiography. However, MRI accessibility and the need of sedation relegate its use to selected cases in which diagnosis is not clear with less demanding techniques. These include differentiating transient synovitis from a septic arthritis or osteomyelitis [30], diagnosis of inflammatory joint disease or bone tumors, and early detection and follow-up of Perthes disease.

MRI findings correlate with prognosis in LCPD. These include extent and distribution of epiphyseal necrosis, subchondral ossified nucleus fracture, involvement of the lateral pillar, and disturbance of physeal growth, including presence of transphyseal neovascularity or bridging [31].

Recent studies have been focused on the role of diffusion weighted MRI because it does not need contrast medium administration. ADC ratio of the femoral metaphysis was positively correlated with the Herring classification. ADC ratio superior to 1.63 indicates bad prognosis with 89% sensitivity and 58% specificity [32].

In adult patients, MRI is currently playing a definite role in the assessment of osteoarthritis. Although traditionally belonging to the arena of radiographs, the role of MRI has been stressed after the term femoral acetabular impingement was coined in 2003 [33]. Growing interest has been focused in accurate diagnosis of the acetabular and femoral morphological abnormalities that may lead to early osteoarthritis.

MR imaging is considered paramount to these objectives, mainly when surgery is considered, due to the ability of MRI to portray the whole section of the femoral neck surface, as well as to image the labrum and articular cartilage.

Diagnosis of impingement can only be achieved if, besides imaging findings, there are also clinical symptoms and positive impingement maneuvers [34].

**Figure 9:**
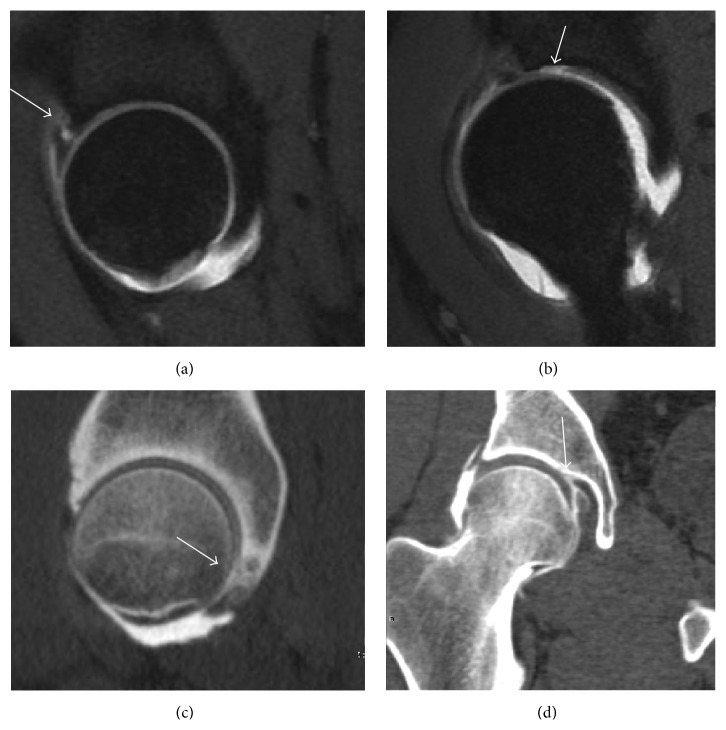
Sagittal T1 weighted images showing anterosuperior labral tear (a) and chondral lesion (b). Sagittal CT-arthrography showing posteroinferior chondral injury (c) and coronal CT-arthrography (d) showing ligamentum teres tear.

Most of the angles and measurements described in the plain radiograph section can be accurately reproduced on MRI. In addition, the superiority of MRI resolution with intra-articular contrast allows detection of labral and chondral abnormalities that may influence the choice of medical, percutaneous, or surgical management (Figure 9).

MR arthrography has proven superior in accuracy when compared to native MR imaging. It is considered the best technique to assess the labrum. Knowledge of the normal variable morphology of the labrum helps to differentiate tears from normal variants. A triangular shape is most commonly seen in 66% of asymptomatic volunteers, but round, flattened, and absent labra can also be found in asymptomatic populations [35]. MR arthrography has demonstrated sensitivity over 90% and specificity close to 100% in detecting labral tears. Loose bodies are demonstrated as filling defects surrounded by the hyperintense gadolinium [36, 37].

Association between labral tears and chondral damage has been demonstrated. This underscores the interaction between cartilage and labrum damage in the progression of osteoarthritis [38]. Chondral damage to the posteroinferior part of the acetabulum as a contrecoup lesion occurs in approximately one-third of pincer cases secondary to persistent abutment on the anterior part of the joint leading to a slight posteroinferior subluxation. This is considered a bad prognosis sign [39].

MR arthrography can also demonstrate ligamentum teres rupture or capsular laxity, which are debated causes of microinstability of the hip [40]. Elongation of the capsule or injury to the iliofemoral ligament or labrum may be secondary to microtrauma in athletes [41]. MR can demonstrate abnormalities in these cases, such as increased joint volume or a ligamentum teres tear (Figure 9).

Intra-articular osseous causes of pain include several conditions: avascular necrosis (AVN), transient osteoporosis of the hip (TOH), tumors, and stress or insufficiency fractures. All these entities may present with a pattern of bone marrow edema characterized by decreased signal intensity on T1 weighted images and increased signal intensity on fluid sensitive sequences, such as fat saturated T2-weighted or STIR images. When there is no evidence of a focal lesion associated with the edema pattern, TOH is suspected [42]. When a band of low intensity is seen inside the edematous area, the shape and length of this band become important. It is generally convex to the articular surface in the case of subchondral stress or insufficiency fractures, whereas it is concave, circumscribing all of the necrotic segment, in cases of AVN. When doubts do persist, gadolinium-enhanced MRI tends to show that the proximal portion beyond the band is enhanced in fractures but is not in AVN [43].

**Figure 10:**
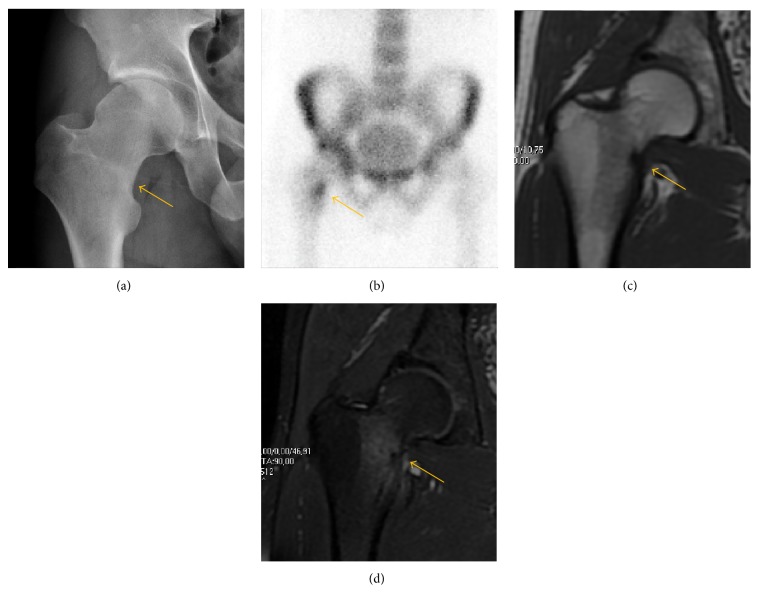
Stress femoral neck fracture in a young athlete barely visible in X-ray film as a sclerotic line (arrow) (a). Tc 99 scintigraphy shows a band of uptake (b), while T1 (c) and DP fat saturated (d) weighted MR images showed the fracture line and a pattern of edema.

MRI has been shown to have 100% sensitivity and specificity in prospective studies of occult hip fractures. These fractures were diagnosed by bone marrow edema and a low signal fracture line, mainly on T1 or T2 weighted images [44] (Figure 10).

In synovial proliferative disorders, MRI demonstrates synovial hypertrophy. In the case of PVNS, characteristic foci of low signal intensity related to hemosiderin deposition are better seen on gradient echo T2^*∗*^ images [45] (Figure 7). In the case of synovial osteochondromatosis, the synovial hypertrophy is accompanied by intermediate signal cartilaginous loose bodies and/or low signal calcified loose bodies [46].

## 6. Computed Tomography

Due to radiation concerns, CT has been relegated after MRI in the study of intra-articular causes of hip pain. The only exception where CT is considered superior to MRI is in bone tumors, because of its ability in characterizing matrix calcifications, and in depicting the anatomy of acute traumatic fractures. Typical matrix calcifications include the following: (a) osteoid mineralization, like a dense cloud, (b) chondroid calcification, reproducing a punctate popcorn pattern, or (c) fibrous calcification, ground glass-like appearance. There are also tumors that typically do not show matrix calcification. CT is also used for accurate localization of the nidus in osteoid osteomas and this must be differentiated from Brodie's abscess or a stress fracture [47]. The current standard treatment of osteoid osteoma is percutaneous radiofrequency ablation and this is usually performed under CT guidance [48].

Quite often, CT is widely available unlike MRI, especially in the acute setting. CT is performed in this setting when doubt about the existence of a fracture persists following plain radiograph. Modern multidetector computed tomography (MDCT) shows results comparable with MRI for detecting occult fractures [49].

Due to the submillimeter resolution of MDCT arthrography, many authors consider this technique complementary to MR arthrography. It may even have superior sensitivity in detecting cartilage pathology, but lesser detecting labral tears [50, 51].

CT can also be used to obtain accurate measurement of the femoral version and torsion. The femoral version is measured by an angle formed between a line through the femoral head-neck axis and another horizontal line drawn between both ischial tuberosities. Normal values range between 5 and 25°. Retroversion is considered abnormal.

Femoral torsion is the angle between a line along the femoral head and neck axis and a second line that is touching the posterior border of both femoral condyles. The normal value at birth is approximately 32° and decreases gradually with age. In adults, the normal value ranges from 10° to 20° [52].

## 7. Ultrasound

Ultrasound is the first-choice technique for diagnosis of newborns hip dysplasia. In experienced hands with appropriate technology, ultrasound can also be useful during the first year of life [53]. Some European healthcare systems encourage universal ultrasound screening in neonates between the sixth and eighth weeks. Although it shows higher initial costs caused, it leads to significant reduction in the total number and overall costs of dysplastic hips undergoing operative and nonoperative treatment [54, 55].

**Figure 11:**
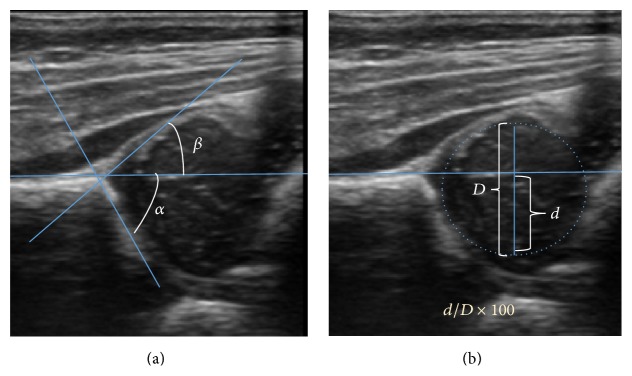
(a) Useful ultrasound measures in neonatal hip sonography, alpha and beta angles. (b) Measurement of femoral head coverage.

Ultrasound allows categorizing pediatric hips, according to Graf's criteria, in four main types: normal, immature, and dysplastic (subluxed and dislocated) [56]. This classification is based on measurements of the acetabular inclination angle (alpha), cartilage roof angle (beta), and infant age [57]. The femoral head coverage can also be determined by dividing the length of the femoral head covered by the acetabular fossa and the diameter of the femoral head. Its lower normal limits are 47% for boys and 44% for girls [58] (Figure 11).

In a recent study, including newborns with high clinical suspicion for DDH [59] (Ortolani/Barlow test, asymmetry in abduction of 20° or greater, breech presentation, leg-length discrepancy, and first-degree relative treated for DDH), hip sonography led to a change in clinical diagnosis in 52% of hips and to a change in management plan in 32% of hips. It obviated further follow-up in 23%, strengthening its role as an important technique reassuring the clinical diagnosis [60].

During childhood, ultrasound is a quick method to assess hip pain and quite often may be used to avoid use of irradiating techniques, such as radiography or CT. Ultrasound allows evaluation of joint effusion, synovial thickening and neovascularity, the bone/cartilage contour, and the femoral head-neck alignment. Although sonography is extremely sensitive in detecting increased synovial fluid, it is nonspecific and cannot be used with accuracy to determine the type of fluid. Transient synovitis of the hip, despite being the most frequent cause of pain in children between 3 and 10 years, remains a diagnosis of exclusion. It usually shows anechoic fluid, but echogenic fluid can also be found [61]. The effusion is considered pathologic when it is measured at >2 mm in thickness [62]. The differential diagnosis is wide, including osteomyelitis, septic arthritis, primary or metastatic lesions, LCPD, and SCFE [63]. Discrimination from septic arthritis is challenging, often requiring joint aspiration. In septic arthritis, US is able to demonstrate a hip joint effusion, synovial thickening, and cartilage damage, although the appearances are nonspecific [64].

**Figure 12:**
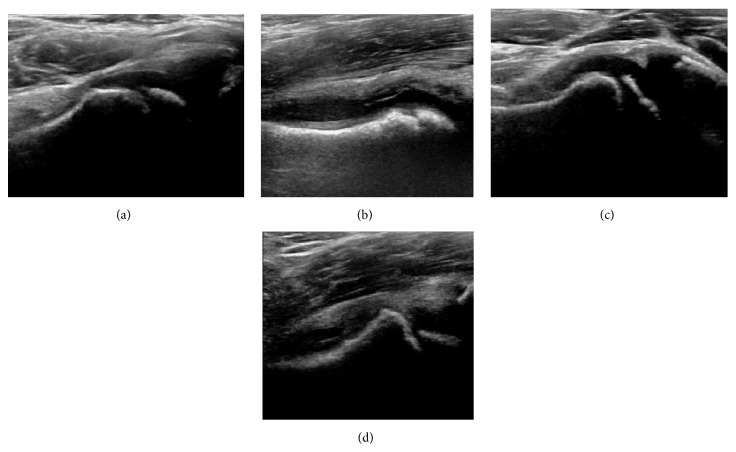
(a) Normal ultrasound appearance of the femoral head-neck junction. (b) Joint effusion in transient synovitis of the hip. (c) Flattening of the femoral head in a patient with Perthes disease. (d) Step in the femoral head-neck junction in a patient with SCFE.

A step between the head and the physis can be detected in children with SCFE, while abnormalities in the femoral head contour may suggest the presence of LCPD. In both cases, radiographs are mandatory to confirm diagnosis and severity [65] (Figure 12).

In adults, the most common application for US is to detect tendon or muscle injuries, effusion or synovitis within the hip joint or its adjacent bursae [66]. Joint effusions may be due to many intra-articular processes and this may need another imaging technique to achieve a specific diagnosis.

## 8. Nuclear Medicine

Bone scanning in patients with hip pain can be complementary to other imaging studies, mainly in indeterminate bone lesions to clarify whether it is an active lesion with abnormal radiotracer accumulation. Nevertheless, MRI has replaced scintigraphy in the diagnosis of most of these conditions. An example is stress or insufficiency fractures: increased uptake is usually present in around 80% of fractures within 24 h, and 95% of fractures reveal activity by 72 h following trauma [67], showing an overall sensitivity of 93% and specificity of 95% [68]. MRI is superior to bone scans in terms of sensitivity (99%-100%) and specificity (100%) [69, 70]. Moreover, a bone scan does not provide detailed anatomical location of the fracture, and further imaging is usually required [71].

## Figures and Tables

**Table 1:** Measurements in adult hip.

Measurement	Measure	Normal value
Acetabular depth ratio	Deepness of acetabulum	>250
Center-edge angle	Coverage of acetabulum	20–40
Tönnis angle	Slope of the sourcil	0–10°
Sharp angle	Acetabular slope	<45°
Crossing ratio	Percentage of acetabular walls crossing	<20%
Alpha angle	Degree of bulging of the femoral head-neck junction	Male > 68° Female > 50°
Femoral head-neck offset	Offset of the femoral head with regard to most prominent aspect of the femora neck	>10 mm
Offset percentage	Femoral head-neck offset related to femoral head diameter	>0.18
